# Tuberculosis of the rare azygos lobe of the right lung

**DOI:** 10.1016/j.rmcr.2021.101424

**Published:** 2021-05-11

**Authors:** Abdelrhman Abo-Zed, Mohamed Yassin, Tung Phan

**Affiliations:** a Department of Medicine, University of Pittsburgh Medical Center, Pittsburgh, PA, USA; b Division of Clinical Microbiology, University of Pittsburgh and University of Pittsburgh Medical Center, Pittsburgh, PA, USA

**Keywords:** Mycobacterium, Tuberculosis, Azygos lobe, Right lung, Biopsy

## Abstract

*Mycobacterium tuberculosis* is the etiological agent of tuberculosis in humans. Tuberculosis is not only a highly contagious infectious disease, but also one of the top causes of death globally, especially in developing countries. Tuberculosis generally affects the lungs, and tuberculosis of the azygos lobe of the lung is extremely rare. This case report presents such a unique finding.

## Highlights

• *Mycobacterium tuberculosis* causes tuberculosis in humans.
• Tuberculosis is a highly contagious infectious disease.
• Tuberculosis of the rare azygos lobe of the right lung is reported.

## 1 Introduction

*Mycobacterium tuberculosis* causes tuberculosis (TB), one of the top causes of death globally, claiming 1.7 million lives back in 2016 [1]. It is no mystery that *Mycobacterium tuberculosis* can survive not only in a high-oxygen environment but also in the hypoxic environment within necrotic granulomas [2]. Pulmonary tuberculosis can produce a wide range of symptoms and signs. Pulmonary consolidation accounts for 50% of radiographic abnormalities, followed by mediastinal lymphadenopathy (35%) and cavitation (29%). Literature review suggests that 13–30% of pulmonary TB cases present without upper lobe infiltrates or have atypical radiographic patterns [3]. Of these atypical radiographic patterns, tuberculosis of the azygos lobe is extremely rare. Here we report the usual case of tuberculosis in the azygos lobe of the right lung in an immunocompetent individual.

## 2 Case presentation

**Fig. 1:**
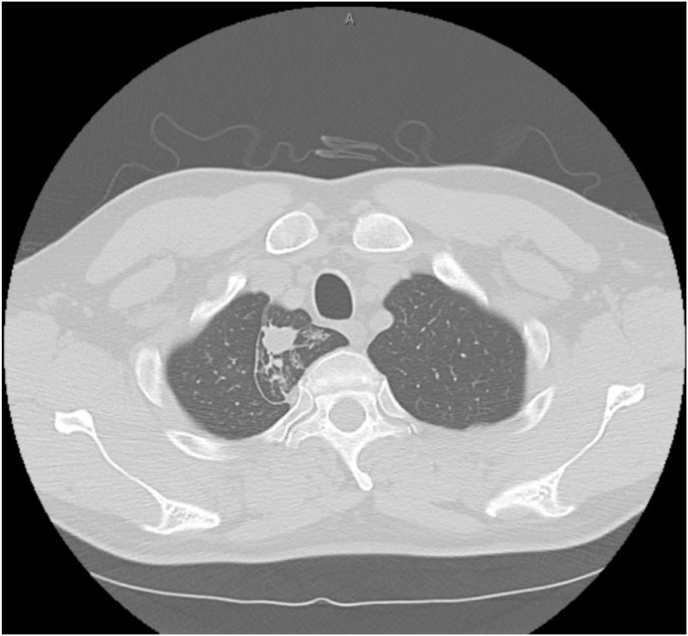
A computed tomography (CT) scan of the chest showing multiple nodules, including in the azygous lobe, right upper lobe, and right lower lobe, with associated micronodular patterns.

A 53-year-old male with a history of alcohol abuse, anxiety and insomnia presented to an outpatient clinic for his annual physical examination. He smoked half a pack of cigarettes a day for 25 years. Currently he drinks beer 2–3 times per week, but heavy liquor more frequently in the past. He denied illicit drug use and high-risk sexual activities. He was active and independent with activities of daily living. During the visit, the patient was complaining of coughing in the morning, bringing up small amounts of sputum, and of dyspnea upon exertion for some time. The patient was afebrile, and had blood pressure of 125/80 mmHg, heart rate 60 bpm. Physical exam did not have any rhonchi or wheezes. All other systems were negative. His white blood cell count was 6700/μL with 52% neutrophils, 33% lymphocytes. A chest x-ray was ordered and revealed a questionable ill-defined medial right upper lobe infiltrate, which could be related to pneumonia. However, no sputum was collected for further evaluation. There were no pleural effusions or pneumothorax. Subsequently, a computed tomography (CT) scan without contrast of the chest showed right upper and lower lobe tree-in-bud micro-nodularity in a *peri*-bronchovascular distribution. There were associated larger nodular opacities in the right upper lobe measuring up to 2.0 × 1.2 cm at the right apex. The patient was prescribed Levaquin for seven days, and the QuantiFERON-TB Gold test was ordered. The patient was advised to have a follow-up visit after one month. At that time, the patient felt much better and had minimal cough. However, his pulmonary function showed mild chronic obstructive defect without significant bronchodilator reversibility. The repeat CT scan of the chest showed multiple nodules in the right upper and lower lobes, especially in the azygos lobe. All these nodules were associated with a branching micronodular pattern (Fig. 1). In addition, the patient's QuantiFERON-TB Gold test was positive. The bronchoscopy was performed and showed a normal appearing endobronchial anatomy. The right lower lobe had normal findings. Attention of the scope was focused to the apex of the right lung. Multiple transbronchial lung biopsies were taken from the azygos lobe as well as the atypical subsegment of the right upper lobe. Microbiological studies revealed *Mycobacterium tuberculosis* in both the bronchoalveolar lavage and lung biopsy specimens. Tissue specimens were also sent for histopathology showing necrotizing granulomatous inflammation. The patient started on ethambutol, rifampin, pyrazinamide and isoniazid with vitamin B6 supplementation for two months, followed by rifampin and isoniazid with vitamin B6 for six months. Patient had been asymptomatic and no side effects so far at a follow-up visit a couple of months later.

## 3 Discussion

*Mycobacterium tuberculosis* is a species of pathogenic bacteria in the family Mycobacteriaceae, and it was first discovered in 1882 by Robert Koch [4]. This bacterium is an aerobic, slow growing nonmotile bacillus, and it has a waxy lipid-rich outer wall that contains high concentrations of mycolic acid [5]. In this case, the bronchoalveolar lavage and lung biopsy specimens taken during the bronchoscopy were sent to the clinical microbiology laboratory for further evaluation. Mycobacterium was grown in MGIT media. Buff colored colonies with a dry bread-crumb-like appearance were seen in Lowenstein-Jensen slant. Ziehl-Neelsen stain was performed, and many long acid-fast bacilli were found. The final identification of *Mycobacterium tuberculosis* was confirmed by the Gen-Probe AccuProbe culture identification test (DNA probe).

*Mycobacterium tuberculosis* causes tuberculosis in humans, and this disease remains a global health problem [6]. In this case, the chest x-ray showed infiltrate in the right upper lobe. Subsequently, the patient got the CT scan of the chest, which showed infiltrative lesions. He was started on fluoroquinolone (Levaquin) while waiting for the QuantiFERON-TB test's result. Drug resistance of mycobacterium tuberculosis has emerged, and the continuing spread of drug-resistant tuberculosis is one of major public health challenges [7,8]. In 2013, appropriately 480,000 new cases of multidrug-resistant tuberculosis were reported [9]. The susceptibility testing was performed against several antibacterial agents (pyrazinamide, isoniazid, ethambutol, and rifampin), and the mycobacterium isolate in this case was susceptible to all of them. While tuberculosis may infect any part of the body, it primarily affects the lungs [10,11]. However, tuberculosis of the azygos lobe in the lungs is extremely rare. The azygos lobe is a rare anatomical variant, most often encountered in the right lung, and its prevalence in the clinical setting is 0.4% [12]. Up to date, there are only two reports about tuberculosis of the azygos lobe [13,14]. Our study is the third case presenting such a unique finding.

## Author contributions

AA: Investigation, Writing - original draft; TP and MY: Writing - review & editing.

## Ethical statement

Approval from the ethical committee was not required due to the nature of this case report. Abiding by the Declaration of Helsinki, patient anonymity was guaranteed.

## 4 Submission declaration and verification

The work described has not been published previously. It is not under consideration for publication elsewhere, and its publication is approved by all authors.

## Funding sources

This case study did not receive any funding.

## Declaration of competing interest

The authors declare that they have no conflict of interest.

## References

[1] Koch A., Mizrahi V. (2018). Mycobacterium tuberculosis. Trends Microbiol..

[2] Bartek I.L., Woolhiser L.K., Baughn A.D., Basaraba R.J., Jacobs W.R., Lenaerts A.J., Voskuil M.I. (2014). Mycobacterium tuberculosis Lsr2 is a global transcriptional regulator required for adaptation to changing oxygen levels and virulence. mBio.

[3] Woodring J.H., Vandiviere H.M., Fried A.M., Dillon M.L., Williams T.D., Melvin I.G. (1986). Update: the radiographic features of pulmonary tuberculosis. AJR Am. J. Roentgenol..

[4] Cambau E., Drancourt M. (2014). Steps towards the discovery of Mycobacterium tuberculosis by Robert Koch, 1882. Clin. Microbiol. Infect..

[5] Brennan P.J. (2003). Structure, function, and biogenesis of the cell wall of Mycobacterium tuberculosis. Tuberculosis.

[6] Amdekar Y.K. (2005). Tuberculosis -- persistent threat to human health. Indian J. Pediatr..

[7] Willcox P.A. (2000). Drug-resistant tuberculosis. Curr. Opin. Pulm. Med..

[8] Maitre T., Aubry A., Jarlier V., Robert J., Veziris N., CNR-MyRMA (2007). Multidrug and extensively drug-resistant tuberculosis. Med. Maladies Infect..

[9] Dousa K.M., Kurz S.G., Bark C.M., Bonomo R.A., Furin J.J. (2020). Drug-resistant tuberculosis: a glance at progress and global challenges. Infect. Dis. Clin..

[10] Yadav J., Verma S., Chaudhary D., Jaiwal P.K., Jaiwal R. (2019). Tuberculosis: current status, diagnosis, treatment and development of novel vaccines. Curr. Pharmaceut. Biotechnol..

[11] Golden M.P., Vikram H.R. (2005). Extrapulmonary tuberculosis: an overview. Am. Fam. Physician.

[12] Kotov G., Dimitrova I.N., Iliev A., Groudeva V. (2018). A rare case of an azygos lobe in the right lung of a 40-year-old male. Cureus.

[13] Malaspina M., Gamalero P.C. (1953). A case of tuberculous process of the azygos lobe. Minerva Pediatr..

[14] Karmakar S., Hussain A., Batra S., Prasad K.R.R. (2017). Tuberculosis of the azygous lobe of lung: case report and review of literature. EJMR.

